# Identification of correlated genetic variants jointly associated with rheumatoid arthritis using ridge regression

**DOI:** 10.1186/1753-6561-3-s7-s67

**Published:** 2009-12-15

**Authors:** Yan V Sun, Kerby A Shedden, Ji Zhu, Nam-Hee Choi, Sharon LR Kardia

**Affiliations:** 1 Department of Epidemiology, School of Public Health, University of Michigan, 109 Observatory, Ann Arbor, Michigan 48109, USA; 2 Department of Statistics, University of Michigan, 439 West Hall, 1085 South University, Ann Arbor, Michigan 48109, USA

## Abstract

Using the North American Rheumatoid Arthritis Consortium genome-wide association dataset, we applied ridged, multiple least-squares regression to identify genetic variants with apparent unique contributions to variation of anti-cyclic citrullinated peptide (anti-CCP), a newly identified clinical risk factor for development of rheumatoid arthritis. Within a 2.7-Mbp region on chromosome 6 around the well studied HLA-DRB1 locus, ridge regression identified a single-nucleotide polymorphism that was associated with anti-CCP variation when including the additive effects of other single-nucleotide polymorphisms in a multivariable analysis, but that showed only a weak direct association with anti-CCP. This suggests that multivariable methods can be used to identify potentially relevant genetic variants in regions of interest that would be difficult to detect based on direct associations.

## Background

Rheumatoid arthritis (RA) is an autoimmune disease causing inflammation and soft-tissue swelling of mainly diarthrodial joints. The disease can lead to considerable loss of mobility due to pain and joint destruction. A newly identified autoantibody, anti-cyclic citrullinated peptide (anti-CCP), is strongly correlated with persistent RA and is a better predictor of erosive outcome than rheumatoid factor immunoglobulin M (IgM) [1]. Elevations of anti-CCP have been noted to predict increased risk for development of RA [2]. The HLA-DRB1 shared epitope alleles are strongly associated with the presence of anti-CCP antibodies, and this effect is modulated by HLA-DR3 allele [3].

Complex human diseases such as RA have complicated genetic architectures [4]. Association studies looking at the individual, direct effects of genes suggest that each genetic predictor alone has very weak association with disease status [5]. However, the high heritability identified in many human diseases indicates that the overall genetic contribution to risk is substantial. For complex disease studies, the standard approach of univariable analysis may miss genetic variants whose association with the trait only becomes clear when simultaneously adjusting for the effects of other genetic factors. A natural way to proceed is to apply multiple-regression analysis using a set of genetic variants as the independent variables and the trait as the dependent variable. However, in mapping studies involving dozens of correlated variants, not to mention genome-wide association studies (GWAS), ordinary least square (OLS) regression is far too unstable to be useful due to the high dimension of the independent variable relative to the sample size, and the high correlation among genetic variables caused by linkage disequilibrium (LD).

Ridge regression (RR) [6] was introduced as a method for predictive modeling and for reducing the mean squared estimation error by stabilizing regression estimates in the presence of correlation among the independent variables. RR also provides a means to identify genetic associations along a continuum spanning from direct relationships to multivariable relationships, by changing a tuning parameter. Malo et al. recently proposed using RR to prioritize linked single-nucleotide polymorphisms (SNPs) in genetic association studies according to their unique (potentially causal) associations with the trait [7].

In this study, we explored the ability of RR to identify genetic associations with anti-CCP, a quantitative clinical predictor of RA development. We performed a series of RR analyses using a range of tuning parameters and compared the results with those of single-factor analysis. Focusing on regions of interest around HLA-DRB1 and TRAF1-C5, RR was able to identify a SNP that was strongly associated with anti-CCP variation. This association was strongest in a multivariable analysis accounting for the effects of all other variants in the region, and largely vanished in univariable analysis. The fact that this SNP is not directly correlated with anti-CCP suggests that one or more additional genetic variants in our dataset may also be contributing to the variation of anti-CCP, but that the dataset available to us is not sufficiently large to identify these additional factors with high confidence.

## Methods

### Data

The phenotype and geonotype data provided by the North American Rheumatoid Arthritis Consortium (NARAC) were analyzed, including 868 RA cases and 1194 controls. The analysis dataset includes 867 unrelated RA cases with anti-CCP measured as a quantitative outcome. 545,080 SNPs were genotyped using the Illumina 550 k chip [8] and distributed by Genetic Analysis Workshop (GAW) 16. All available SNPs within a 2.7-Mbp genomic region around *HLA-DR *cluster on chromosome 6 and the 100-kbp *TRAF1-C5 *region on chromosome 9 [8] were used in this study. SNPs with minor allele frequency (MAF) smaller than 0.05 were excluded, leaving 957 SNPs. These SNPs were then subjected to an iterative procedure in which a randomly selected SNP in the most correlated pair of SNPs was dropped. This procedure was iterated until no SNP pairs correlated more than 0.7 remained, which left us with 328 SNPs.

### Statistical methods

Pearson correlation was used for detecting associations between individual genetic variants and anti-CCP, using the Fisher transform and the asymptotic standard deviation to form a *Z*-score for the null hypothesis of no direct association. Population genetic parameters for all SNPs were calculated, including MAFs, genotype frequencies, and either a chi-square test or the Fisher exact test for departures from expectations under Hardy-Weinberg equilibrium (HWE).

### Ridge regression

In RR, the estimated coefficients of the linear model solve a penalized least-squares criterion with the penalty being proportional to the squared L2-norm of the coefficients: , where *Y *is the *n*-vector of trait values, *X *is the n × m design matrix whose *i*^th ^row contains the genotype of sample *i *on genetic variants *j *= 1,2,...,*m*, *β *is the *m*-vector of effect estimates for the genetic variants, and *λ *is the tuning parameter. The genetic marker and trait data are centered so no intercept is included when fitting the regression. The expected value of  is , where *F*_*λ *_= *X'X *+ *λ I *and *M *= *X'X*. The covariance matrix of  is . Therefore, it is possible to calculate numerically the vector of *Z*-scores . When *λ *= 0, the *Z*-score is the same as what would be obtained using OLS. When *λ *→ ∞, the *Z*-scores converge to values that are proportional to the univariable Pearson correlation coefficients between each independent variable and *Y*.

A complicating factor is that when *λ *> 0, the *Z*-score *Z*_*λi *_need not have mean zero under the specific null hypothesis *β*_i _= 0 - this is only guaranteed under a global null hypothesis of no effects among all modeled genetic variants. However, for small values of *λ*, the degree to which the *Z*-scores are off-center is small, and can be accommodated by re-standardizing the *Z*-scores. This is accomplished by considering a quantile-quantile plot of the observed *Z*-scores against corresponding standard normal quantiles. Based on an assumption that the middle 90% of the *Z*-scores correspond to zero effects, we fit a simple linear regression to the points on the quantile-quantile plot. The slope of this line estimates the standard deviation of the null *Z*-scores, which tends to be slightly greater than 1 due to the lack of centering. By scaling the *Z*-scores with the reciprocal of this standard deviation, any lack of centering of the null *Z*-score means is presumed to be eliminated.

Our main interest is to identify genetic variants associated with the trait that do not show direct associations in univariable analysis. We will do this by considering RR *Z*-scores for several nonzero values of *λ*. The *Z*-scores are then re-standardized as described above, and univariable *Z*-scores are also calculated. Bonferroni corrections of the *Z*-scores are used to account for multiple comparisons. Randomization of the trait vector *Y *is used to assess whether the overall significance level is maintained after considering the results for several *λ *values.

## Results

### Identification of masked effects using RR

**Figure 1:**
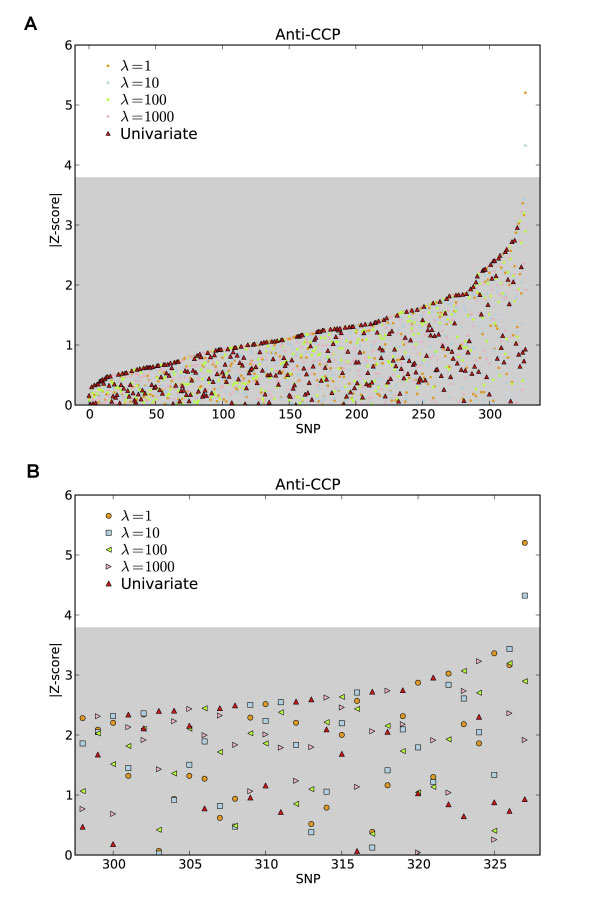
**Results of RR analysis and univariable analysis in identifying SNPs associated with anti-CCP**. The SNPs are ordered by the maximal value of the absolute *Z*-score. The gray box shows the null region based on Bonferroni correction for one test per SNP. A, All SNPs; B, SNPs with the largest *Z*-scores.

The *Z*-scores for 328 SNPs in the *HLA-DR *and *TRAF1-C5 *regions were calculated using RR with tuning parameters *λ *= 1, 10, 100, and 1000. We found that coefficient estimates and *Z*-scores varied continuously with *λ*, and these values are representative of the results that occur for other *λ *values. As expected, the *Z*-scores were slightly over-dispersed, with standard deviations estimated from their central values ranging from 1.02 to 1.1. The *Z*-scores were then rescaled by the reciprocals of these factors. The *Z*-scores based on Fisher-transformed Pearson correlation coefficients were also calculated for each SNP. For each value of *λ*, the SNPs were ranked in absolute value and compared to a Bonferroni threshold of 3.8. The maximal *Z*-scores among the univariable model and the four RR models (*λ *= 1, 10, 100, and 1000) were used to rank the 328 SNPs (Figure 1). A single SNP (rs2844533) was significant for *λ *= 1 and *λ *= 10, with a *Z*-score exceeding 5 for *λ *= 1. This SNP had a *Z*-score smaller than 1 (insignificant) in univariable analysis of anti-CCP, and its *Z*-score varied smoothly with a high value exceeding 5 (when *λ *= 1). No SNPs were significant for larger values of *λ*, or for the univariable analysis. SNP rs2844533 is located at 31,458,781 bp on chromosome 6, about 25 kbp upstream of *HLA-B *gene, and about 1 Mbp downstream of *HLA-DRB1 *gene.

Randomization of the trait vector, followed by the same analysis as performed with the actual trait values, produced *Z*-scores exceeding the Bonferroni threshold in approximately 1 in 20 data sets, as expected (data not shown). This suggests that our re-standardization was successful at creating statistics that are well approximated by a standard normal null distribution. We repeated the analysis under Box-Cox transforms of the trait value of the form (y^*κ*^-1)/*κ*, and found that for *κ *ranging from 1 (no transformation) to 0.55 (approximately a square root transformation), rs2844533 continued to be the top-ranked SNP and was significantly associated with the trait after Bonferroni adjustment.

## Discussion

Even when genes contribute additively to a quantitative trait, the genes having strongest additive effects can often be identified with greater power by using multivariable techniques, rather than scanning for direct associations. A major reason for this is that when several correlated variants are present, each introduces heterogeneity that partially masks the other variants' effects. However, traditional multivariable analysis using OLS regression is ineffective for large numbers of predictor genes, particularly when substantial correlations are present. RR operates on a continuum between univariable analysis and OLS multiple regression, potentially allowing the trade-off between univariable and multivariable analysis to be made optimally in a particular setting. For predictive modeling with a high dimensional and/or substantially correlated covariate vector, RR outperforms OLS regression [9]. It can also handle data with *p *> *n *(i.e., number of predictors is larger than the sample size).

Here we used RR to identify a strong association in a mildly ridged, multiple regression fit (*λ *values from 1 to 10 correspond to effective degrees of freedom of 280 to 326, compared to 328 for OLS regression) focusing on a candidate region of interest extracted from a GWAS data set. As demonstrated here, an important application of RR will be to help in determining whether multiple disease loci are located in a candidate region following a genome-wide scan, such as the strong LD region within 15q25.1 associated with lung cancer [10].

Sample size is a critical issue when using multiple regression analysis for genetic mapping, particularly in the case of a complex disease where individual genetic variants only explain a small portion of total variance [5]. In principle, with a sufficiently large sample OLS can be used to identify the unique contributions of the markers in a region. Here we demonstrate, consistent with other reports, that RR can be used to identify multifactorial genetic associations in situations where multiple regression fit by OLS and univariable analysis both fail to identify any associations. In genetic mapping using several hundred correlated genetic variants, RR appears able to partially reveal the complicated genetic structures. Further development may extend the utility of RR to even higher dimensional data sets, and to categorical traits.

## Conclusion

By considering a sequence of RR analyses and rigorously calibrating the estimates of genetic effects, we identified a single SNP showing a strong association with a quantitative trait related to RA. The most plausible explanation for this finding is that multiple genetic variants in our data set are contributing to anti-CCP variation in a way that obscures the direct relationships between genetic variants and the trait. While univariable analysis and multiple regression fit using least squares yielded no associations with rigorous significance levels, the RR procedure allowed us to identify one SNP with high confidence. In this case we were unable to identify the other genes presumed to be associated with anti-CCP variation; however, our inability to identify these synergistic factors does not detract from the potential importance of the SNP we did find.

## List of abbreviations used

CCP: Cyclic citrullinated peptide; GAW: Genetic Analysis Workshop; GWAS: Genome-wide association studies; HWE: Hardy-Weinberg equilibrium; IgM: Immunoglobulin M; LD: Linkage disequilibrium; MAF: Minor allele frequency; NARAC: North American Rheumatoid Arthritis Consortium; OLS: Ordinary least square; RA: Rheumatoid arthritis; RR: Ridge regression; SNP: Single-nucleotide polymorphism.

## Competing interests

The authors declare that they have no competing interests.

## Authors' contributions

YVS and KAS participated in the design and coordination of the study, performed the statistical analysis, and drafted the manuscript. JZ, N-HC and SLRK participated in the design of the study. All authors read and approved the final manuscript.
